# The Experience of Patients with Schizophrenia Treated with Repetitive Transcranial Magnetic Stimulation for Auditory Hallucinations

**DOI:** 10.1155/2013/183582

**Published:** 2013-06-02

**Authors:** Priya Subramanian, Amer Burhan, Luljeta Pallaveshi, Abraham Rudnick

**Affiliations:** ^1^Department of Psychiatry, Regional Mental Health Care-London, Western University, London, ON, Canada N6A 4H1; ^2^Lawson Health Research Institute, London, ON, Canada N6C 2R5; ^3^Department of Psychiatry, University of British Columbia, Vancouver Island Health Authority, Victoria, BC, Canada V8R 4Z3

## Abstract

*Introduction*. Auditory hallucinations are a common symptom experience of individuals with psychotic disorders and are often experienced as persistent, distressing, and disruptive. This case series examined the lived experiences of four individuals treated (successfully or unsuccessfully) with low-frequency (1 Hz) rTMS for auditory hallucinations. *Methods*. A phenomenological approach was used and modified to involve some predetermined data structuring to accommodate for expected cognitive impairments of participants and the impact of rTMS on auditory hallucinations. Data on thoughts and feelings in relation to the helpful, unhelpful, and other effects of rTMS on auditory hallucinations, on well-being, functioning, and the immediate environment were collected using semistructured interviews. *Results*. All four participants noted some improvements in their well-being following treatment and none reported a worsening of their symptoms. Only two participants noted an improvement in the auditory hallucinations and only one of them reported an improvement that was sustained after treatment completion. *Conclusion*. We suggest that there are useful findings in the study worth further exploration, specifically in relation to the role of an individual's acceptance and ownership of the illness process in relation to this biomedical intervention. More mixed methods research is required to examine rTMS for auditory hallucinations.

## 1. Introduction

Auditory hallucinations, that is, the experience of sound in the absence of external perceptual stimuli, are a common symptom of individuals with psychotic disorders such as schizophrenia and schizoaffective disorder. The prevalence of auditory hallucinations in schizophrenia is reported to be 75% [1]. Many of these individuals experience auditory hallucinations as persistent, distressing, and disruptive. Up to 30% of individuals with auditory hallucinations respond only partially or not at all to standard antipsychotic treatment [2, 3]. Shergill et al. [4] concluded in their review of psychological treatments for auditory hallucinations that, although these are of some benefit, they often result in improvement in the auditory hallucinations-associated distress, or control over the experience, rather than in elimination or reduction in auditory hallucinations. 

Low-frequency (1 Hz) repetitive transcranial magnetic stimulation (rTMS) in the left temporoparietal cortex (TPC) has been used for the treatment of refractory auditory hallucinations, with some success. This area has been targeted because previous neuroimaging research suggests that activation of the bilateral superior temporal regions may have a role in its genesis [5, 6]. The low frequency (1 Hz) of rTMS produces sustained reductions in cortical activation [5]. It has also been shown to affect functional connectivity of the targeted region (temporo-parietal junction) and general psychopathology [6, 7]. Gromann et al. [8] concluded that normalizing the functional connectivity between the temporoparietal and frontal brain regions may underlie the therapeutic effect of rTMS on auditory hallucinations in schizophrenia. Despite consistent application of rTMS to the targeted area, results are variable. A meta-analysis of studies evaluating the effect of rTMS on the left TPC as treatment for auditory hallucinations suggested a significant positive effect for rTMS [9] but a more recent meta-analysis [10] has reported a lower effect size for TMS on the left temporoparietal cortex for auditory hallucinations. It is unclear whether there are potential disorder-related and person-related factors that may predict responses to rTMS treatment for auditory hallucinations. We report here a case series to explore these predictors and generate related hypotheses. 

The current paper describes the experiences of four people treated with rTMS for auditory hallucinations related to schizophrenia, using guided first-person accounts. The research question addressed was as follows:

 what is the lived experience of people who were treated (successfully or unsuccessfully) with rTMS for auditory hallucinations?

## 2. Method

The sample consisted of four participants who had previously received treatment with rTMS for auditory hallucinations, that is, 1 Hz TMS applied over the left TPC (localized through the 10-20-20 EEG system) by figure-of-eight air-cooled coil using the Magstim Superrapid II machine for 5 daily sessions per week (1600 pulses per session) for 2 weeks. The study was informed using a phenomenological approach [11], modified to involve some predetermined data structuring to accommodate for expected cognitive impairments of participants, with a focus on lived experiences in relation to the rTMS intervention and its impact on auditory hallucinations.

Participants who were treated with rTMS were interviewed within 4 weeks from the last day of treatment with rTMS. Participants recruited for this study met the following inclusion criteria: (1) age of 18 years or more; (2) a primary diagnosis of schizophrenia with auditory hallucinations; (3) treatment with rTMS for auditory hallucinations at the authors' institution, completed in the past 4 weeks. 

The Hallucinations Change Scale (HCS) was not part of the study but was used as a tool for monitoring the outcome of rTMS treatment in the authors' institution. The HCS is a self-rated visual analogue scale personalized for each individual based on a narrative description of auditory hallucinations prior to treatment commencement. The baseline is set by definition to 10 and subsequent descriptions are scored between 0 and 20 with 0 = no hallucinations and 20 = hallucinations twice the severity of the baseline experience. Visual analogue scales, although less useful for assessing interindividual differences, are sensitive to changes in each individual [12] and have shown satisfactory reliability and sensitivity to change in the area of rTMS treatment of auditory hallucinations [5, 13]. The ratings on the HCS were collected as data to determine successful or unsuccessful outcome from treatment. “Successful treatment” was defined as a 50% reduction in auditory hallucinations, that is, a score of 5 or less on the HCS, and “unsuccessful treatment” was defined as no reduction, a reduction less that 50%, or a worsening of auditory hallucinations. Participants were excluded if they refused to provide informed consent or have inability to provide consent. All treating psychiatrists, along with the involved mental health care teams, were informed about the study and were able to refer potential participants for involvement in the study. 

All four participants provided informed consent to the study. This study was approved by the authors' academic centre's Human Research Ethics Board.

Data collection consisted of two parts: (1) individual semistructured interviews with predetermined prompts; and (2) chart reviews to collect data required for the clinical-demographic form (CDF).

A semistructured interview was conducted with each participant individually. The interview guide addressed the following issues: thoughts and feelings in relation to the helpful, unhelpful, and other effects of rTMS on auditory hallucinations; thoughts and feelings in relation to the helpful, unhelpful, and other effects of rTMS on well-being, functioning and the immediate environment; and thoughts about causation of these effects. 

All the semi-structured interviews were audio recorded and transcribed verbatim and validated by research staff. Identifying confidential information was removed from all the transcripts. Participants' transcripts were used for the analysis, which was informed by case study research [11, 14]. 

## 3. Results

Table 1 lists the HCS scores for each participant before rTMS treatment commencement (Day 1) and after rTMS treatment completion (Day 10). Three out of four participants showed a reduction in the HCS scores to 50% or less indicating successful treatment.

The qualitative data obtained from individual interviews is summarized below.

*Participant* 1. Mr. A is a 37-year-old male who received rTMS treatment for his schizophrenia-related auditory hallucinations. He reported a relaxing effect from the repeated tapping and described that the treatment felt like a “mental massage.” He stated that he did not have any long-term benefits from rTMS but felt better for 30–60 minutes after the treatment. He was not sure as to why the affects wore off so quickly after the treatment. In relation to the negative effects of rTMS, Mr. A noted a slight discomfort in swallowing due to the need to stay still while reclining. He stated that there was no worsening in his psychiatric symptoms or functioning, and that there were no other negative effects. He stated that overall the treatment had neither positive nor negative effects on his auditory hallucinations, well-being, functioning, and environment. He did not find it cumbersome that he had to travel to the hospital every day during treatment.

*Participant* 2. Mr. B is a 54-year-old male who has been diagnosed with schizophrenia and cooccurring obsessive compulsive disorder. He experienced distressing auditory hallucinations which impact his day-to-day functioning. He received rTMS as treatment for his auditory hallucinations. He stated that he experienced a slight discomfort from the tapping sound of the machine but became used to it after one or two sessions. Mr. B reported that the rTMS treatment helped him cope and better understand his illness. Although the rTMS did not completely eliminate the auditory hallucinations, he stated that these hallucinations were more manageable and his thoughts were clearer as a result of rTMS. Before rTMS, he felt that his hallucinations were very strong and at times frightening as well as made it difficult to make decisions due to the distraction. Mr. B felt that there were no negative effects of the treatment but reported an increase in his obsessive compulsive disorder symptoms. He described that after rTMS he began to obsess about cleanliness and engaged in washing his hands more. He was not sure as to whether this was a direct result of the treatment itself. He stated that he could concentrate better and was less distracted by the auditory hallucinations. He also reported feeling happier and more alert to his surroundings. Overall, Mr. B maintained that the treatment was a success for him and that it took repeated sessions (at least three sessions out of a total of ten planned) and a large amount of hope and positive thinking before any noticeable results (i.e., auditory hallucinations becoming quieter) occurred. He also stated that the mere knowledge that the TMS treatment was available should the auditory hallucinations become unmanageable allowed him to “fight the voices” better. 

*Participant* 3. Mr. C is a 32-year-old male who has been diagnosed with schizophrenia and has been noted to experience auditory hallucinations by his family and service providers. Mr. C received rTMS at the request of his mother who reported significant improvement in distractibility following a previous rTMS treatment for his auditory hallucinations. Mr. C was interviewed with his mother present (at his request). Mr. C expressed no discomfort during the rTMS treatment. He reported that the rTMS helped make his thoughts more clear. Mr. C realized that rTMS treatment was given for auditory hallucinations but claimed that he did not experience any auditory hallucinations before or after rTMS. Mr. C reported that he did not experience any negative effects or changes in his well-being, functioning, or environment as a result of rTMS. Mr. C's mother reported an improvement in him talking to himself and in his concentration.

*Participant* 4. Mr. D is a 45-year-old man who has been diagnosed with schizophrenia and experiences auditory hallucinations. Mr. D has received rTMS as treatment for persistent auditory hallucinations. Mr. D expressed that the rTMS helped him relax more during the treatment. He stated that listening to the repeated tapping helped him move his focus away from the auditory hallucinations. He felt that the treatment decreased his auditory hallucinations slightly. Overall, he felt that the rTMS was a good experience due to the relaxation he felt in his mind and body. Mr. D postulated that there may be no predictors and that TMS may just randomly help some people while not others (stating that all treatments work for some and not for others). Mr. D also expressed that rTMS had no negative effect on his well-being, functioning, or environment. 

## 4. Discussion and Conclusion

All participants reported here were adult men between the ages of 30 years and 55 years. They were all considered treatment refractory and were on clozapine. There were no changes to the treatment in the two months preceding, during, or one month following the low-frequency TMS treatment. Three out of the four participants in the study self-reported auditory hallucinations. One participant (Participant 3) denied ever experiencing auditory hallucinations although history from his clinical chart documented the presence of auditory hallucinations that considerably disrupted his social and occupational functioning and which improved significantly during and after the course of TMS. All four participants noted some improvements in their well-being following treatment: Participants 1 and 4 reported relaxation, Participant 2 reported happiness, improved alertness, and hope, and Participant 3 reported increased clarity of thought. However, only Participants 2 and 4 noted an improvement in the auditory hallucinations (which was corroborated by improved HCS scores at the end of treatment), and only Participant 2 reported an improvement that was sustained after treatment completion. These findings are similar to those of a systematic review by Poulet et al. [15], which suggests that an acute course of low-frequency rTMS significantly reduces auditory hallucinations but that the effects are typically not long-lasting (8.5 weeks on average). Qualitative and quantitative data sometimes conflict, which can prompt the generation of hypotheses and further study. Such a discrepancy was found in the case of patient number 3, who reported no hallucinations at baseline in his interview but reported a reduction of his hallucinations post-rTMS on the HCS. This difference in reporting of baseline hallucinations on his part may be explained by the fact that his mother was present when he was interviewed, which may have caused him to deny his hallucinations, possibly as part of dissimilation behavior, which sometimes occurs in families with psychopathology [16]; reporting on the HCS is less detailed and hence may have facilitated his disclosure of presumed baseline hallucinations on it. This explanation raises the possibility that standardized one-item reporting may provide valid data even when compared to qualitative data, which could be further explored in other research; it is already recognized that they can provide reliable data [17]. Also, removing family from such interviews as a condition of participation could be considered, although we uphold participant choice in that and other matters. 

Overall, none of the participants reported a worsening of their well-being or symptoms. While Participants 1 and 4 reported the tapping to be a positive experience, Participant 2 reported that it was a discomfort. Importantly, Participant 2 reported a worsening in his obsessive compulsive symptoms. There are several case reports and retrospective studies in relation to the worsening of obsessive compulsive disorder symptoms due to antipsychotic medications [18], including clozapine [19]. It is thought that this worsening may be related to the effects of antipsychotics on the dopamine system [20]. A recent meta-analysis of rTMS by Slotema et al. [21] noted that rTMS in the treatment of the negative symptoms of schizophrenia, using a 10 Hz frequency stimulation to the left dorsolateral prefrontal cortex (DLPF) [22–24] and 1 Hz frequency stimulation to the right DLPF [25], produced several side effects including an increase in the symptoms of comorbid obsessive compulsive disorder. However, rTMS is being studied as an intervention to treat OCD with promising results [26–28].

Participant 2 noted that having repeated sessions, being hopeful, and keeping a positive view of the treatment helped with the treatment process. While a placebo effect cannot be completely ruled out, we believe that some of the benefits that this participant experienced may have been related to an awareness of his illness experience and an acceptance of some of the manifested symptoms, notably the auditory hallucinations. He also appeared to be committed to seeing an improvement in these symptoms and to be taking control of the recovery process using the rTMS as a tool or strategy to cope with the illness. Learning to regulate activity, taking the role as active agents, and being determined and hoping to overcome the illness and to cope with their symptoms help people with schizophrenia regaining a sense of responsibility over their life [29].

Such use of biomedical treatment as part of mastery of illness has been shown to be effective in other patient populations, such as people with diabetes mellitus, who feel and function better when they are in control of their insulin injections.

However, there are limitations to this study. As a case series comprising a small sample, its findings cannot be robustly generalized. Also, the challenges in recruitment with this population [30] were compounded by the limited availability of rTMS as a treatment option at the authors' institution. This may have inadvertently led to a skewed sample of individuals who were favourably biased toward rTMS. Nevertheless, we posit that there are useful findings in the study worth further exploration, specifically in relation to the role of an individual's acceptance and ownership of the illness process in relation to this biomedical intervention as well as in relation to the effects of rTMS on cooccurring syndromes such as obsessive compulsive disorder which is commonly comorbid with schizophrenia.

## Figures and Tables

**Table 1:** Distribution of the Hallucination Change Score (HCS) obtained from patient's chart review.

Study participants	Day 1 of rTMS	Day 10 of rTMS
Participant 1	10	10 (U)
Participant 2	10	5 (S)
Participant 3	10	2 (S)
Participant 4	10	4 (S)

U: unsuccessful, S: successful.
